# The teaching and learning of communication skills for social work students: a realist synthesis protocol

**DOI:** 10.1186/s13643-022-02125-w

**Published:** 2022-12-12

**Authors:** Emma Reith-Hall

**Affiliations:** Department of Social Work and Social Care, School of Social Policy, Muirhead Tower, Edgbaston, Birmingham, England

**Keywords:** Realist review, Realist synthesis, Social work education, Social work students, Communication skills

## Abstract

**Background:**

Good interpersonal communication is the cornerstone of social work practice, enhancing the outcomes of people in receipt of its services. Social workers’ communication skills are often identified as an area of concern. Communication skills can be developed and refined through training or education. The teaching and learning of communication skills is firmly embedded in many social work qualifying courses; however, considerable heterogeneity exists regarding such complex interventions and the theoretical underpinnings of which have not been made explicit. Realist synthesis can help explain how, why, for whom and in what circumstances an intervention might work, which is an important first step for helping educators to tailor courses to meet the needs of different learner groups and, where applicable, the employing agencies and government departments who fund them.

**Methods:**

Realist synthesis is an interpretive, theory-driven and explanatory approach that aims to explain the interplay between the context, mechanisms and outcomes of interventions. This realist synthesis seeks to understand and explain to what extent, how, why, for whom and in what circumstances complex educational interventions aimed at teaching communication skills to social work students produces its effects. A five-step process will be followed iteratively. In step 1, the initial programme theory will be developed. Step 2 will involve searching for evidence. In step 3, selection and appraisal will take place. Step 4 requires data to be extracted and organised, and in step 5, data will be analysed and synthesised.

**Discussion:**

The teaching and learning of communication skills in social work education is under theorised. The findings from this realist synthesis aim to help policymakers and educators make informed decisions about the design and delivery of complex educational interventions aimed at improving the communication skills of social work students. The realist synthesis will be conducted and reported in accordance with the RAMESES guidelines and standards.

**Systematic review registration:**

The review is registered with the Open Science Framework. https://doi.org/10.17605/OSF.IO/BYHC7

**Supplementary Information:**

The online version contains supplementary material available at 10.1186/s13643-022-02125-w.

## Background

There is considerable consensus within the literature that good interpersonal communication is the cornerstone of social work practice [1, 2], enhancing the outcomes of people in receipt of its services [3]. Serious case reviews and commissioned reports commonly identify social workers’ communication as an area of concern. Since interpersonal communication is a goal-driven and goal-directed process ‘undergirded by perceptual, cognitive, affective, and behavioural operations’ [4], communication skills can be developed and refined through training or education. Communication skills are firmly embedded within the curriculum of social work qualifying courses in a number of different countries including Australia, the UK and the USA [5–7]. In the UK, teaching communication skills became mandatory following the introduction of the degree programme 20 years ago [8]. The content, sequencing and pedagogy underpinning the educational interventions were not prescribed; hence, considerable variation exists both within the UK and further afield.

Knowledge and practice reviews have identified that the outcomes evidence underpinning these interventions is limited, and that the theoretical underpinnings of the teaching and learning of communication skills have not been made explicit [9–11]. Some time has passed since these reviews were undertaken, during which considerable research activity has taken place and new routes into the profession have proliferated. In the UK, for example, Think Ahead and Frontline seek to recruit high-achieving graduates, whilst Step Up and the new social work apprenticeship degrees recruit experienced support staff into undergraduate programmes. The time is ripe to revisit the literature on the teaching and learning of communication skills in social work education to update our knowledge so that policy and practice decisions can be better informed.

To address the first gap within the literature — the outcomes evidence — a systematic review aimed at investigating whether or not the teaching and learning of communication skills is effective has recently been undertaken [12, 13]. Notwithstanding significant methodological challenges, there was overall consistency in the direction of mean change for the development of communication skills of social work students following training [13]. To address the second gap within the literature — the need to theorise the intervention — a broader range of study designs is required which can explain how and why interventions might work [14, 15]. Realist synthesis is particularly suited to this purpose since programme theories help explain how the intervention is supposed to work. Preliminary searching indicates that the body of evidence has grown in the last two decades, suggesting that fresh insights into the mechanisms underpinning communication skills courses in social work education should be reinvestigated. The explanation a realist synthesis can provide about how, why and for whom an intervention might work is an important first step for helping educators tailor courses to meet the needs of different learner groups and, where applicable, the employing agencies and government departments who fund them.

The review protocol is registered on the OSF database (https://doi.org/10.17605/OSF.IO/BYHC7).

## Methodology

### Realist synthesis

Realist synthesis is an interpretive, theory-driven approach [16] which reviews different types of information, evidence and literature about complex social interventions. Methodological inclusivity and pluralism are encouraged. Realist synthesis applies a realist philosophy of science, that is an external (real world) reality exists, but this can only be understood through human interpretation (senses, language and culture) ‘to the synthesis of findings from primary studies’ ([15], p.93) that aims to explain causation within interventions through context-mechanism-outcome configurations.

The realist approach recognises that no theory can always explain or predict the outcomes of a complex social intervention in every context. Whilst programmes provide opportunities and resources, the outcomes are ultimately determined by the choices and decisions of its participants. Yet, the realist approach assumes that because only a limited number of options are available in any given context, individuals are likely to, though will not always, make similar choices about the resources they use. In realist terms, these semi-predictable reoccurring patterns of behaviour are known as ‘demi-regularities’ [16]. Realist synthesis seeks to ‘uncover the underlying theories that explain these demi-regularities by critically scrutinising the interaction between context, mechanism and outcome in a sample of primary studies’ [17], which are commonly expressed as ‘context–mechanism–outcome configurations’ (‘CMOCs’). Mechanisms, defined as ‘underlying entities, processes, or structures which operate in particular contexts to generate outcomes of interest’ [18], are a defining feature of realist research. They help us understand that it is not the intervention itself which produces outcomes but people’s reactions, reasoning and responses to it that are important.

In realist research, the relationship between context, mechanism and outcome is explored through a variation of the question, ‘What works, for whom, in what circumstances, in what respects and why?’ From this, ‘the reviewer constructs one or more middle-range theories to account for the findings’ ([15], p. 94). Through an iterative process, realist synthesis seeks ‘to gradually develop and refine the programme theory so that it is more detailed, realist in nature and the inferences within it are supported by data’ [19]. In later stages of the inquiry, following a series of different iterations, a number of C-M–O configurations are developed and then tested, using the data gathered in the review. The configurations seek to explain in which context(s) and which mechanism(s) are ‘triggered’ to produce which outcomes(s). The refined realist programme theory should be in the ‘middle range’, that is it should be specific enough to permit empirical testing but abstract enough to provide useful explanations transferable to other situations where the same mechanisms may be operating.

The realist approach is particularly suited to education research, where multicomponent interventions are complex and outcomes are highly context dependent and influenced by the reactions, responses and reasoning of both educators and learners. In relation to medical education, Wong et al. (2012, p. 90) [15] explain that ‘the impact of the “same” intervention will vary considerably depending on who delivers it, to which learners, in which circumstances and with which tools and techniques'. The same point can be made about social work education generally and the teaching and learning of communication skills more specifically. It is for this reason that a realist approach was deemed appropriate for investigating this topic.

### Objectives

The intended objectives of this realist synthesis are as follows:

1. To understand and explain to what extent, how, why, for whom and in what circumstances the teaching and learning of communication skills for social work students produces its effects. Theory adjudication (determining which theories best explain the intervention) and cross-case comparison (comparing how the intervention works for different groups or in different settings) will be investigated, provided sufficient detail is included within the primary studies.
2. To provide recommendations, based on the above explanation, and to help educators make informed decisions about the design and delivery of pedagogic practices.

### Review questions

1. To what extent does the teaching and learning of communication skills for social work students produce its intended outcomes?
2. What formal substantive theories are used to inform the teaching and learning of communication skills for social work students?
3. What are the mechanisms by which the teaching and learning of communication skills for social work students are believed to result in their intended outcomes?
4. What are the important contexts which determine whether the different mechanisms produce their intended outcomes?
5. In what circumstances are such interventions likely to be effective?

These questions, deemed important by key stakeholders, will be iteratively refined once the exultant literature is better understood. Whilst question 1 has been addressed by the aforementioned systematic review [12, 13], the author wonders whether additional outcomes, and a greater understanding of the complexity of the intervention, might be demonstrated through a broader range of study designs. Questions 4 and 5 may not be answerable through existing studies and may need to be addressed subsequently, through realist evaluation.

### Ethical considerations

Ethical approval was not required for this synthesis because the literature is in the public domain.

### Synthesis structure and features

**Fig. 1:**
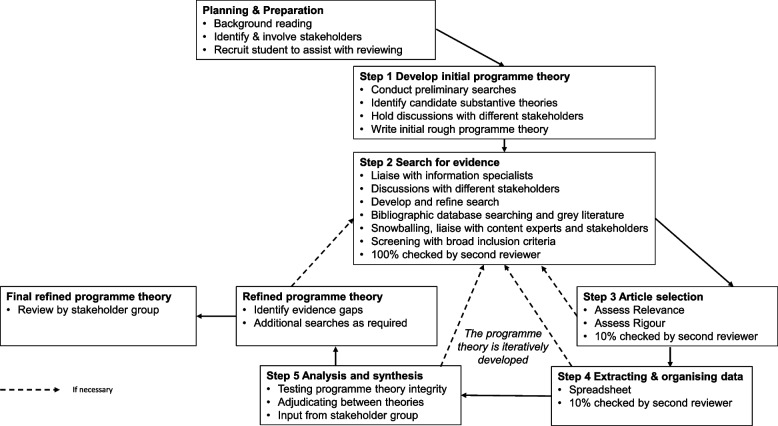
Steps of a realist synthesis

The synthesis is informed by Pawson’s (2006) five stages (identifying the review question, searching for primary studies, quality appraisal, extracting the data, synthesising the data and disseminating the findings) [16]. An initial explanatory theory will be developed, after which the ‘programme theory’ will be tested and refined against data from empirical studies. A visual representation, informed by Duddy and Wong’s (2018) depiction [20], which outlines the approach underpinning this realist synthesis, is provided in Fig. 1 below.

#### Planning and preparation

##### Background reading

As a researcher, who is also an academic in the field, I regularly read and review the literature for teaching, research and other scholarly purposes to ensure that my content knowledge and expertise are current and relevant. Through sustained immersion, familiarisation with the literature was already well established before this particular research project began.

##### Identifying and involving stakeholders

Citing Ryan and Hood (2004) [21] and Schwandt (2005) [22], Suri and Clarke (2009) [14] suggest that ‘the knowledge construction of educational practices can be considered incomplete and oppressive if it undermines the rich knowledge of different stakeholders, especially teachers and students, whose practices and experiences are the sites for educational research’ (p. 412). In realist research, identifying and involving stakeholders is encouraged from the outset. In addition to policymakers, the key stakeholders involved in social work education are students, academics, practitioners, and people with lived experience (sometimes referred to as service users and carers). My commitment to, and experience of, collaboration and partnership working [23–25] supports my ability to work with different stakeholder groups, using their ‘lived experience’ and/or content expertise to focus the review and inform the development and refinement of the programme theory. Deviating slightly from the more established practice of bringing different stakeholder groups together, I have met different stakeholder groups separately. This was partly to ensure that the voices of more powerful groups do not become privileged above less powerful groups and also for more practical reasons — getting everyone together in one place has not been feasible when there is no funding available to reimburse expenses and was not deemed responsible in light of a global pandemic given the existing health conditions of some collaborators. Using a reflexive approach, I, as the researcher, will consider whether this strategy needs to be adapted as the research project progresses. Stakeholders will be involved throughout the research process, as shown in Fig. 1.

#### Step 1 — Develop the initial programme theory

Realist inquiry begins (and ends) with a programme theory [20]. The initial programme theory tends to operationalise a set of assumptions of the programme designers about how the programme is expected to work. Preliminary literature searches and stakeholder consultations allow the programme theory to be iteratively developed and help determine the priorities of the realist synthesis. Discussions with stakeholders have influenced the nature and form of this realist synthesis. For example, the lack of a coherent theoretical framework to inform the teaching and learning of communication skills [9] is an issue of particular interest for social work academics, which influenced the decision to place more emphasis on identifying candidate substantive theories within the literature. Formal substantive theories ‘provide a bridge to a wealth of existing research and knowledge about a topic’ and operate at a higher level of abstraction than programme theories [26].

### Preliminary searches

A series of preliminary scoping searches aimed at retrieving substantive theories from the literature have been undertaken. The first of the preliminary searches entailed searching the Social Care Institute of Excellence (SCIE) website for any grey literature sources by selecting ‘communication skills’ from the subject topic menu of the resources and services section. Two more structured searches were also undertaken: a database search of the Web of Science and a discipline-specific journal search of *Social Work Education*, the *British Journal of Social Work* and the *Journal of Social Work Education*. The searches were guided by the BeHEMoTh (behaviour of interest, health context, exclusions, model, theory) approach [27], using various terms to describe the behaviour of interest (communication/interpersonal), adapting the health context (social work education) alongside the suggested terms for theory or model (theor*/model*/framework*, concept*). No exclusions were applied, and the theory concepts were not restricted to title and abstract as it was anticipated that information about theories might be located in the main text and reference lists.

**Fig. 2:**
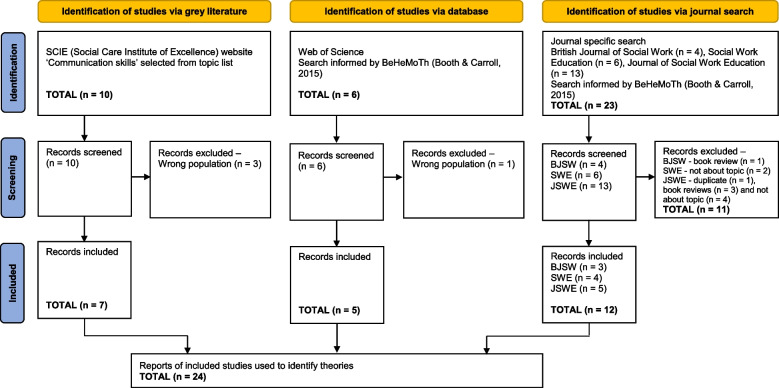
PRISMA 2020 flow diagram for preliminary searches to identify substantive theories

The combined searches produced a total of 39 records. Ten grey literature resources were retrieved from the SCIE website, including the aforementioned knowledge reviews. Six records were retrieved through the web of science search and twenty-three through the discipline-specific journal search (4 from *Social Work Education*, 6 from the *British Journal of Social Work* and 13 from the *Journal of Social Work Education*). Each record was added to an EndNote group folder. Fifteen records were excluded for the following reasons: duplication (*N* = 1), the record was a book review (*N* = 4), the topic was not about the teaching and learning of communication skills (*N* = 6) and the population did not comprise social work students (*N* = 4). The remaining 24 records were read in full. A PRISMA flow diagram of the preliminary searches is depicted in Fig. 2.

### Reference and citation tracking

Recognising that theory might be contained within a sibling paper, reference and citation tracking were undertaken manually and using Google Scholar, which led to the identification of two additional records.

### Discussions with stakeholders

Following the preliminary searches, discussions with stakeholders took place regarding substantive theories. Contact was made with social work academics involved in the teaching and/or researching of communication skills. Their content expertise confirmed that relevant candidate theories had been identified.

### Substantive theories

The key substantive theories underpinning the teaching and learning of communication skills which were explicitly referred to in the literature found through the theory search outlined above are included below:

- Experiential learning theory
- Reflective practice
- Adult learning theory
- Theory of living human systems
- Relational/cultural theory
- The postmodern and post-structural approach
- Task-centred and behavioural approaches
- Humanistic/person-centred counselling approaches, including microskills training
- Psychosocial theory

Experiential learning theory and humanistic person-centred approaches were most frequently mentioned in the studies identified through the theory search, a finding supported by a recent systematic review [13]. Experiential learning theory synthesises the contribution of scholars, including educational psychologists and philosophers, who positioned experience as playing a fundamental role in learning, training and educational development of adult learners who bring their personal and professional experiences with them. Experiential learning involves learning by experience, in which the learner takes on an active role, followed by reflection and analysis of that experience, which further develops their learning.

Kolb’s (1984) experiential learning cycle [28] was the most frequently cited reference to theory in the records identified through the preliminary searches, followed by Donald Schön’s (1983) [29] work on reflective practice and Knowles’ (1978) [30] adult learning theory. These theories are associated with a constructivist view of education, ideas that can be traced back to John Dewey.

Ivey and Authier’s (1971, 1978) [31, 32] microskills approach provides a systematic method for teaching beginning communication skills to counsellors and therapists. It shares similarities with the above theories, although its roots lie in psychotherapy, particularly in humanistic person-centred counselling approaches, developed by Carl Rogers (1951, 1961) [33, 34]. The other theories were mentioned in just one study. These included Agazarian’s (1997) system-centred therapy for groups [35], Miller and Stiver’s (1997) ‘relational/cultural theory’ [36] and Jessup and Rogerson’s (1999) postmodern and post-structural approach [37]. A brief overview of the substantive theories identified through the preliminary searches are provided in Table 1.

**Table 1:** Overview of substantive theories

*Kolb’s (1984) experiential learning theory*
Rooted in the intellectual traditions of social psychology, philosophy and cognitive psychology, Kolb saw learning as the process, whereby knowledge is created through the transformation of experience. Kolb’s theory of experiential learning proposes that people learn best when they are engaged in first-hand experiences which can then be reflected on to inform future practice. Reflection, a key instrument in experiential learning, was identified by Kolb as one of the mechanisms through which experience could be transformed into knowledge, skills and attitudes. Experiential learning theory encourages educators to create learning experiences which are sequential, progressively developmental and provide learners with opportunities to reflect on their experiences
*Schön’s (1983) reflective practitioner*
Schön (1983) viewed self-reflection as a vehicle for learning and improvement. He distinguished between reflection in action and reflection on action. He observed that professionals reflect in action by applying their knowledge within any given situation and then adjusting their practice accordingly. Practitioners use hindsight to reflect on action, thinking through how they could improve future practice. These processes enable practitioners to master increasingly complex, uncertain and challenging situations
*Knowles’ (1978) adult learning theory*
Knowles’ (1978) adult learning theory takes account of the vast amount of practical experience which adult learners possess. Self-concept, the role of experience, readiness to learn, orientation for learning, internal motivation and understanding why knowledge is required are important concepts in adult education. Developing the idea of andragogy — the art and science of helping adults to learn — adult learning theory highlights the importance of creating a learning environment which enables adults to feel accepted, respected and supported. A spirit of mutuality and trust between students and facilitators as joint enquirers is deemed helpful for adult learners
*Humanistic/person-centred counselling approaches, including microskills training*
Person-centred counselling and interviewing draw on ideas from humanistic psychology which proposes that human beings have the potential to overcome distress and work towards self-actualisation within the context of a facilitative helping relationship. Carl Rogers (1951, 1961) introduced core conditions of helping relationships which included the demonstration of congruence, empathy and positive regard Specific models which derive from person-centred approaches include the following: • The microskills approach (Ivey and Authier, 1978) [31, 32] • The human relations training model Carkhuff and Truax (1965) [38] and Carkhuff (1969) [39] • The skilled helper model (Egan, 2021) [40]
*Communication theory*
Communication theory, influenced by systems thinking, looks at how information is exchanged, including how messages are conveyed, received and acted upon
*Task-centred and behavioural approaches*
Task-centred and behavioural approaches to skills acquisition provide systematic opportunities for the development of basic skills including students interviewing each other or service-user educators within workshops and skills labs [41]
*Psychosocial approaches*
Influenced by Freud’s psychoanalytic theory, psychosocial approaches consider how concepts such as internal mental processes (the ‘psyche’), group dynamics and ‘use of self’ impact on communication
*Agazarian’s (1997) system-centred therapy for groups*
According to Koprowska (2003, 296) [42], system-centred therapy ‘deliberately structures group norms in order to reduce restraining forces and increase driving forces’. The part played by ‘predictable defences’ needs to be ‘undone’ so that new information can be integrated in ways that enable students to ‘move away from personal preoccupations toward a process of professional discovery’ (Koprowska, 2003, 306) [42]
*Miller and Stiver’s (1997) ‘relational/cultural theory’*
Based on the work of Jean Baker Miller and Janet Stiver, ‘relational/cultural theory’ suggests that ‘how students are taught will influence what they learn, and that this in turn will influence how they use this knowledge and understanding in practice’ [9]. In the context of social work education, the ideas of mutual engagement, mutual empathy and mutual empowerment are just as important in the student–teacher relationship as they are in the client-worker relationship
*Jessup and Rogerson’s (1999) postmodern and post-structural approach*
Described as a discourse and practice, the postmodern and post-structural approach argues that interpersonal communication in social work must integrate personal and socio-structural domains. The teaching and practising of interpersonal communication skills are located within a political context

The theories are not mutually exclusive, and there is significant overlap between them. The role of the substantive theories will be considered in the realist synthesis.

### Development of programme theory

From the combined preliminary searches and discussions with stakeholders, an initial programme theory was developed.

A wide range of teaching and learning activities were identified, which involved combining formal input (on theory or background) from an instructor with experiential or practice-based activities such as group exercises, group discussions, role-plays, simulations and skills laboratories, video work, observation, feedback and reflection. Service user and carer involvement and shadowing experienced social workers in practice were also identified within the literature identified through the preliminary searches. The different activities and components that communication skills courses comprise contributed to a ‘theory of action’. Some of the resources also described how the intervention is thought to work. Pedagogic methods that encouraged learning by doing and reflection were common, explaining why assessment, evaluation, feedback and reinforcement were frequently commented on in the literature.

In terms of the intended programme outcomes, the studies referred to confidence [43, 44] and interpersonal communication skills [45]. Knowledge and attitudes were also mentioned within the domains of ‘knowing’ and ‘being’ [46]. Discussions with different stakeholder groups consulted during the development of the systematic review protocol suggested that self-efficacy, knowledge, attitudes and skills were the outcomes of importance [12, 13] and are captured within Carpenter’s (2005) framework for social work educational outcomes [47].

**Fig. 3:**
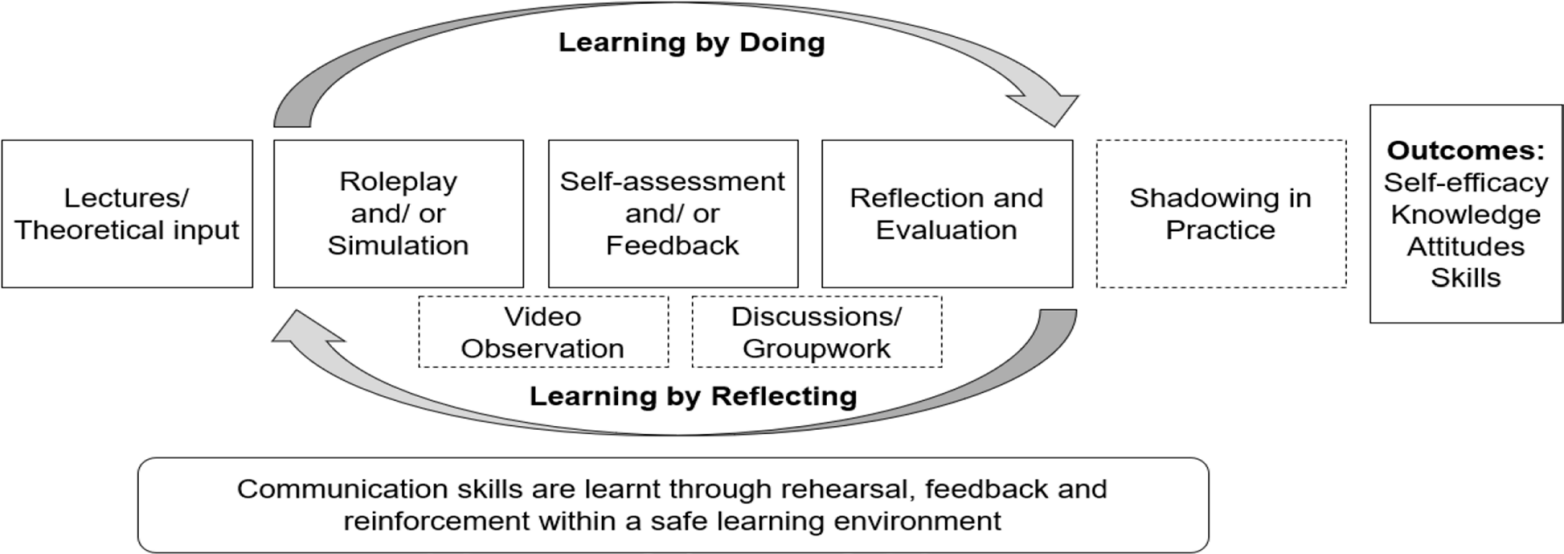
Initial rough program theory

Pawson (2006, p. 74) [16] suggests that the initial rough theory should contain some key features of realist explanation, comprising ‘conjectures on the generative mechanisms that change behaviour, ideas on the contexts that influence its operation, and hunches about the different outcome patterns that ensue’. Successful outcomes appeared to be dependent upon students engaging in ‘learning by doing’ and ‘learning through reflection’, tenets which are supported by two of the substantive theories (experiential learning theory and reflective practice). A safe learning environment was deemed to be an important context for the learning by doing component to take place. Figure 3 shows the initial rough programme theory.

From the studies identified through the preliminary search and discussions with stakeholders (students, people with lived experience and social work academics), it was also possible to identify some tentative and provisional context-mechanism-outcome configurations, as stated below, where C = context, M = mechanism and O = outcome.

1. In a safe learning environment [C], students will experience a sense of trust [M] and manage performance-related anxiety, fear and embarrassment [M], enabling them to engage [M] in practice opportunities to improve their communication skills [O].
2. Students are more likely to demonstrate effective communication skills [O] when practice scenarios are authentic [C] because they perceive it to be believable [M] and/or useful [M], which motivates them [M] to perform well.
3. In the context of supportive and constructive working relationships [C], students will take feedback on board [M], evaluate [M] and reflect on their skills [M], developing knowledge [O] and confidence [O] to demonstrate communicative improvements [O] in subsequent practice opportunities.
4. In a ‘containing’ and attuned reflective space [C], students will make sense of their own internal worlds and those of others [M], developing self-awareness [O], use of self [O] and emotional capacity [O] to communicate effectively within the helping process [O].

Elements of the first three CMOCs featured repeatedly in the studies. A microskills approach underpinned by humanistic/person-centred counselling, behavioural psychology and experiential learning theory appears to underpin various different participative teaching and learning activities, whereby skills are practised, evaluated and reflected on and then reinforced through further practice. The fourth CMOC, underpinned by a psychosocial approach, appeared to operate at a deeper level and considered how understanding and using ‘the self’ develops in relationships with others. Although this CMOC was most evident in the literature about communicating with children and young people and referred to some very specific activities including tutor modelling, child observation and reflective groupwork, it was possible to glean from the more generic studies that this CMOC would also operate in learning how to communicate with adults. This idea will be tested in the realist synthesis.

Themes relating to power and control also emerged in the studies reviewed for the preliminary search. The role of service user and carer involvement in social work education was highlighted in some of the studies, whereas others considered the importance of students feeling in control of their learning. Although it was not possible to develop a relevant CMOC, this may become possible in the review of the wider body of literature located through the main search.

The purpose of realist research is to ‘gradually develop and refine the programme theory so that it is more detailed, realist in nature and the inferences within it are supported by data’ (Wong, 2015, p. 2) [19]. To support this endeavour, the initial programme theory will be further developed and refined through the subsequent steps in the approach underpinning this realist synthesis, as shown in Fig. 1.

#### Step 2 — Searching for evidence

The main systematic search of the literature aimed at identifying relevant documents and articles from which the programme theory will be developed and tested takes place in step 2. Academics, service users and carers, students and practitioners have been asked for suggestions for key words for the intervention, programme recipients and intended outcomes. An information specialist has helped the researcher formulate the search string for this particular search (based on population and intervention concepts only) using a combination of subject headings and free text, adapted for each database. The basic search string is as follows:

- (“social work student*” OR “student social worker*”)
- AND
- (communicat* OR interpersonal OR interview*)
- AND
- (train* OR educat* OR teach* OR learn* OR curricul*)

Study design and features will not form part of the search criteria of the realist synthesis since ‘nuggets’ of information [48] can be gleaned from quantitative, qualitative and mixed methods research as well as policy documents, opinion pieces and other grey literature. Social science and education databases are less well indexed than those used in medicine and health sciences, so a comprehensive and inclusive search has been constructed. The databases and platforms comprising the formal search are as follows:

- Education Abstracts (EBSCO)
- ERIC (EBSCO)
- MEDLINE (OVID)
- PsycINFO (OVID)
- Web of Science Database Social Science Citation Index
- Social Services Abstracts (ProQuest)
- ASSIA Applied Social Sciences Index and Abstracts (ProQuest)
- ClinicalTrials.gov
- Database of abstracts of reviews of effectiveness
- The Campbell Library
- Cochrane Collaboration Library
- Evidence for Policy Practice Information and Coordinating Centre (EPPI‐Centre)
- Google Scholar — using a series of searches and screening the first 5 pages of results for each search
- ProQuest Dissertations and Theses

Study selection will not be restricted by language, publication date or publication status.

To supplement the systematic search, more emergent techniques of reference and citation tracking will be used, alongside contacting content experts (leading authors and researchers in the field) who will be asked to recommend additional empirical studies or other relevant grey literature.

Documents will be screened by title and abstract (and full text if required) by the researcher and co-reviewer. Discrepancies will be discussed, and a consensus reached, involving other stakeholders if required.

Background searching has revealed that studies tend to focus on one small aspect of the anticipated initial programme theory; therefore, inclusion criteria will be broad, typically containing information about the following:

- Theories (substantive or informal) relevant to learning and teaching communication skills
- Intervention components, e.g. videoing and feedback
- Outcomes (including proximal outcomes) — knowledge, attitudes and values, self-efficacy and skills
- Different student groups, e.g. undergraduate or postgraduate students
- Information about the learning environment and/or relationships including the role of tutors and peers

Exclusion criteria include the following:

- Records that do not relate to an empirical study about a relevant intervention for the teaching and learning of communication skills
- Studies that are not about students on social work qualifying courses
- Studies that focus on learning in placements/practicums only

It is expected that a series of additional searches will take place during the later stages of the review, particularly as gaps in the literature ascertained through the main systematic literature search are identified. Different inclusion/exclusion criteria will apply, and these will be devised iteratively with support from an information specialist. Literature from counselling training and medical education is not currently the focus of this review but may be drawn upon later, to elucidate further information about how particular intervention components work or to explore where similar mechanisms are in operation. Refinements to the programme theory will inform the nature of these additional searches and will be reported in full in the project write-up. The results of each search will be presented using PRISMA style flow diagrams.

#### Step 3 — Selection and appraisal

The researcher will review all of the documents that meet inclusion criteria during screening, assessing them against the two criteria of the following:

- Relevance (i.e. whether there is any information contained within the documents which can be used to support, refute or refine the programme theory)
- Rigour (i.e. whether the data is trustworthy — or not)

Rigour can be difficult to apply in realist research because it is the ‘nugget’ of information [48] which needs to be assessed rather than the methodological quality of the whole study. For example, a methodologically weak study is less problematic if the relevant section for the review is simply a description of the programme’s components compared to using the findings of a study where the internal validity is questionable. Recognising that ‘different types of data will be subject to different judgements of methodological coherence and plausibility’, Duddy and Wong (2018) recommend recording the assessment of each piece of information [20]. This is the approach that will be adopted here to provide a clear and transparent audit trail. Identifying more than one source of data relevant to a programme theory is another strategy suggested by Wong (2018) [49] which will be adhered to at this stage, to enhance trustworthiness. The focus will be on finding sufficient data that is relevant, coherent and supports the aim of developing programme theory. It is intended that student social workers undertaking an evidence-based practice module will be involved in the selection and quality appraisal process, which will also be overseen and supported by the PhD supervisory team.

#### Step 4 — Extracting and organising data

All of the documents from every search will be uploaded into the EndNote reference manager software. Documents which meet the inclusion criteria will be copied into a separate group folder, into which the full-text PDF files will be uploaded. The researcher will extract the main characteristics from each document in the included study group, using a data extraction template. Data from each document will be coded according to the contribution it makes to the developing programme theory. Initially, data will be organised into broad ‘bucket codes’, based on the initial programme theory. The realist logic of analysis developed by Pawson and Tilley (1997) [50] will then be applied. As the data extraction process continues, and the programme theory is gradually and iteratively refined, the data will be recoded and organised into potential C-M–O configurations. The use of data to refine programme theory will be recorded and reported in the project write up. A 10% random subsample of documents will be checked by a second reviewer. Again, discrepancies will be discussed and brought to the attention of an academic acting as an independent adjudicator.

#### Step 5 — Analyse and synthesise data

Realist analysis and synthesis entail ‘juxtaposing, adjudicating, reconciling, consolidating and situating the evidence’ [16], with a view to refining the programme theory. In realist synthesis, the analysis and synthesis of the selected data in step 5 occur concurrently with data selection and appraisal in step 3 and data extraction and organisation in step 4. Through inductive and deductive reasoning, the researcher will move back and forth between the steps, using the data to build and test the CMOCs, iteratively refining the programme theory, as shown in Fig. 1. Additional searches will be conducted as gaps in the literature materialise or where other disciplines can inform our understanding of how particular mechanisms might operate. Stakeholders will be consulted about the development and refinement of programme theory, hopefully adding their own insights and amendments as they see fit. Retroductive reasoning will be used in the later stages, allowing the refinements to programme theory to be ‘made on the basis of what can plausibly be inferred by all the data available’ [20]. The final synthesis will provide an overview of some of the underlying causal mechanisms which are fired in specific contexts to produce particular patterns of outcomes.

#### Dissemination

The dissemination strategy will be developed with stakeholder involvement. Findings will be translated into evidence-based recommendations that can be shared with and applied by policymakers and educators. Findings will also be made available to students and experts by experience.

## Discussion

The teaching and learning of communication skills in social work education is under theorised. This realist synthesis will provide theory-based explanations to determine to what extent the teaching and learning of communication skills in social work education work and for whom, how, why and in what circumstances. The findings from this realist synthesis will help policymakers and educators make informed decisions about the design and delivery of complex educational interventions aimed at improving the communication skills of social work students.

One limitation of this realist synthesis is that it is being undertaken by a PhD student with no recourse to funding stakeholder involvement activities. Although undergradute students may readily take up the screening opportunities, resource constraints will inevitably have an impact on the extent to which stakeholder collaboration evolves. The PhD student’s supervisory team has content and methodological expertise, including conducting systematic reviews and realist syntheses. Their involvement will add further rigour to the conduct of this research.

Another limitation concerns the state of the extant literature. Although there is a reasonable body of literature about the teaching and learning of communication skills in social work education, it is possible that there are gaps, particularly in terms of outcomes and contextual factors, which means some of the research questions might be unanswerable. A comprehensive search will be undertaken with citation and reference harvesting seeking to locate sibling and kinship papers. Authors will be contacted for further information, although now several appear to have research interests in other areas. Stakeholders will be asked to consider any identifiable gaps, which will add to the development and refinement of the programme theory, with the caveat that programme theory can only ever be partial and is of course open to further testing. Despite these limitations, stakeholder interest indicates that a realist synthesis still has much to offer.

Immersion, meticulous data collection, systematic analysis and reflexive thinking are fundamental to the realist approach [15]. Transparency of methods and decision-making is an essential part of realist synthesis to ensure rigour and credibility [20, 51]. To assist this endeavour, the relevant quality and reporting standards and publication standards for realist synthesis will be followed [51–53]. The PRISMA-P statement (included as Additional file 1) has been used to develop this protocol.

## Supplementary Information

**Additional file 1.** PRISMA-P 2015 Checklist.

## Data Availability

Not applicable.

## Abbreviations

CMO: Context, mechanism, and outcome
PRISMA-P: Preferred Reporting Items for Systematic Review and Meta-Analysis Protocols
RAMESES: Realist and meta-narrative evidence syntheses: evolving standards
